# Minimal invasive horizontal ridge augmentation using subperiosteal tunneling technique

**DOI:** 10.1186/s40902-016-0087-8

**Published:** 2016-11-05

**Authors:** Hyun-Suk Kim, Young-Kyun Kim, Pil-Young Yun

**Affiliations:** 1 Department of Oral and Maxillofacial Surgery, Section of Dentistry, Seoul National University Bundang Hospital, 300 Gumi-dong, Bundang-gu, Seongnam City, Gyunggi-do South Korea; 2 Department of Dentistry & Dental Research Institute, School of Dentistry, Seoul National University, Daehak-ro 101, Jongno-gu, Seoul South Korea

**Keywords:** Alveolar bone grafting, Alveolar ridge augmentation, Dental implants, Minimally invasive surgical procedures

## Abstract

**Background:**

The goal of this study was to retrospectively evaluate the prognosis of minimal invasive horizontal ridge augmentation (MIHRA) technique using small incision and subperiosteal tunneling technique.

**Methods:**

This study targeted 25 partially edentulous patients (10 males and 15 females, mean age 48.8 ± 19.7 years) who needed bone graft for installation of the implants due to alveolar bone deficiency. The patients took the radiographic exam, panoramic and periapical view at first visit, and had implant fixture installation surgery. All patients received immediate or delayed implant surgery with bone graft using U-shaped incision and tunneling technique. After an average of 2.8 months, the prosthesis was connected and functioned. The clinical prognosis was recorded by observation of the peri-implant tissue at every visit. A year after restoration, the crestal bone loss around the implant was measured by taking the follow-up radiographs.

One patient took 3D-CT before bone graft, after bone graft, and 2 years after restoration to compare and analyze change of alveolar bone width.

**Results:**

This study included 25 patients and 39 implants. Thirty eight implants (97.4 %) survived. As for postoperative complications, five patients showed minor infection symptoms, like swelling and tenderness after bone graft. The other one had buccal fenestration, and secondary bone graft was done by the same technique. No complications related with bone graft were found except in these patients. The mean crestal bone loss around the implants was 0.03 mm 1 year after restoration, and this was an adequate clinical prognosis.

A patient took 3D-CT after bone graft, and the width of alveolar bone increased 4.32 mm added to 4.6 mm of former alveolar bone width. Two years after bone graft, the width of alveolar bone was 8.13 mm, and this suggested that the resorption rate of bone graft material was 18.29 % during 2 years.

**Conclusions:**

The bone graft material retained within a pouch formed using U-shaped incision and tunneling technique resulted with a few complications, and the prognosis of the implants placed above the alveolar bone was adequate.

## Background

Ridge augmentation techniques are indicated to establish stable placements of dental implant in partial or complete edentulous patients who suffer from insufficient bone volume [1]. For the treatment of an atrophic alveolar ridge, various guided bone regeneration (GBR) methods have been devised, modified, and clinically trialed for their effectiveness [2–4]. In conventional GBR, a flap approach is used via a horizontal incision along the alveolar crest, with two oblique vertical incisions and release of the periosteum. Barrier membranes such as ones made of e-PTFE are often placed upon selection of bone graft materials. In some cases, however, potentially excessive tissue volume results along with the procedural edema and inflammation, exerting tension on the suture line. These procedures could increase the morbidity and discomfort in the patient.

There are few methods to alleviate tension over the suture to prevent membrane exposure. Some literatures suggest use of flap overlapping or different positioning of the flap. In others, they suggest tissue grafting or use of customized suture techniques. However, considering that bone graft material is often overfilled during ridge augmentation to compensate progressive bone resorption, previously mentioned GBR procedures may not provide a solution to resolve excessive tension on the suture line.

Though early membrane exposure does not necessarily result in failure in bone augmentation, it is agreeable that the exposed graft site will influence the prognosis of the graft by inducing infection and ultimately causing bone loss. Machtei reported that early membrane exposure on the GBR around dental implants had a major negative effect on regenerative outcome [5]. Moreover, this excessive tension on the suture line could result in soft tissue dehiscence, which is an undesirable complication especially where esthetics is of concern.

In the early 1980s, minimal invasive horizontal ridge augmentation (MIHRA) using a subperiosteal tunneling technique was suggested by Kent et al. in which a small vertical incision was made in the alveolar ridge and hydroxyapatite particles was injected under the periosteum [6]. The graft showed some success at first, but in studies of the late 1980s, injected hydroxyapatite particles showed instability and a fibrous capsule that prevented partial bone formation was observed [7, 8]. Since then, there has been slow progress in literature studies about MIHRA.

Subperiosteal tunneling technique is a partially blind procedure that requires patience and delicate surgical maneuvers to develop the subperiosteal flap that could form a pocket for graft materials. Though this bone augmentation does not permit a direct view of the deficient ridge, it has advantages of less postoperative complications such as less bleeding, discomfort, bone loss, and surgery recovery time. Moreover, augmentation without application of fixation is still controversial [6–8].

In this retrospective study, prognosis of a minimal invasive horizontal ridge augmentation (MIHRA) using a small labial incision and subperiosteal tunneling method was evaluated.

## Methods

Entire course of study followed guidelines for Good Clinical Practice and was approved by the Seoul National University Bundang Hospital Institutional Review Board (IRB No: B-1607-356-110).

### Patients

A group of 25 partially edentulous patients ranging in age from 18 to 80 years (10 males and 15 females, mean age 48.8 ± 19.7 years), who received bone graft for installation of the dental implants because of alveolar bone deficiency, participated in this retrospective study. All patients signed informed consent forms explaining the purpose of the study. A total of 39 implants were placed in the graft sites. A description of patients and graft sites is shown in Table 1.

**Table 1:** Description of patients and graft sites

	Number	Percent
Patients	25	100
Sex		
Male	10	40
Female	15	60
Age		
Mean	48.8 ± 19.7	
Range	18–80	
Graft sites	25	
Mx. anterior	20	80
Mx. posterior	3	12
Mn. posterior	2	8

### Surgical procedure

**Fig. 1:**
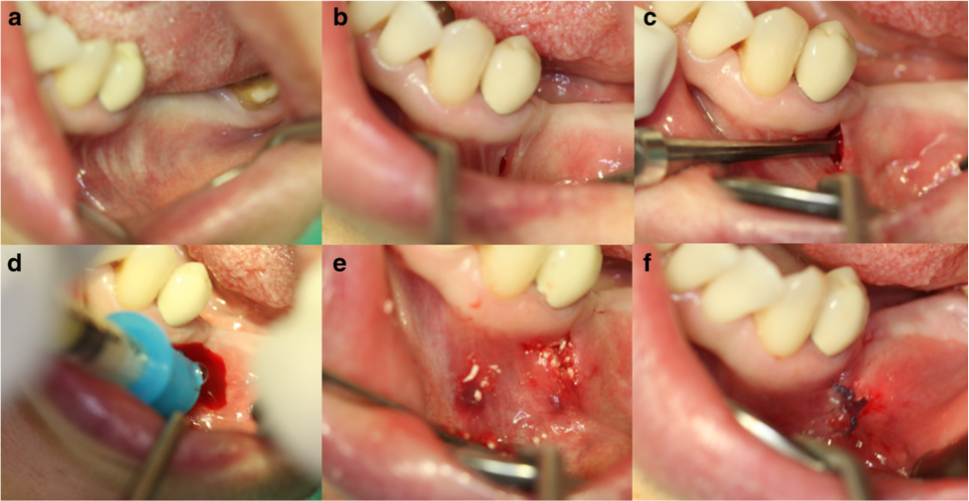
Intraoral photographs of minimal invasive horizontal ridge augmentation using a subperiosteal tunneling technique. **a** Deficient alveolar ridge. **b** Small vertical incision in the buccal side. **c** Preparation of subperiosteal cavity. **d** Bone graft material insertion into the tunnel. **e** After the placement of bone graft material. **f** Closure of the tunnel

All 25 patients received immediate or delayed implant surgery with bone graft using a small buccal incision and subperiosteal tunneling technique. Under local anesthesia using 1 % lidocaine with 1:100,000 epinephrine, a small vertical and/or horizontal incision of less than 10 mm was made in the mucoperiosteum of the labial or buccal sides. A subperiosteal cavity was prepared with a periosteal elevator, and a selection of bone graft materials was placed into the tunnel to augment the deficient alveolar ridge. Various bone graft materials such as autogenous demineralized dentin matrix (ADDM), allograft, and xenograft were selected based upon patients need determined by an experienced oral surgeon. The incision was then closed with 4–0 Vicryl (polyglactin; Ethicon Inc., Sommerville, NJ) using a simple interrupted suture technique. After suture, the graft bone site was molded with a finger to shape appropriate forms (Fig. 1). Patients who underwent surgery took antibiotics (amoxicillin/clavulanate; Augmentin®, Ilsung Pharmaceuticals Co., Seoul, Korea) and a non-steroidal anti-inflammatory drug (talniflumate; Somalgen®, Kunwha Pharmaceutical Co., Seoul, Korea) for 5 days postoperatively. Extra-oral pressure dressing was applied for 3 days to minimize postoperative swelling and 100 mL of 0.1 % chlorhexidine mouth gargling (Hexamedine®, Bukwang Pharm, Ansan, Korea) was prescribed for oral hygiene maintenance. Sutures were stitched out between 10 and 14 days after the surgery and patients were routinely followed every 3 months for clinical and radiological evaluation of implant status.

**Fig. 2:**
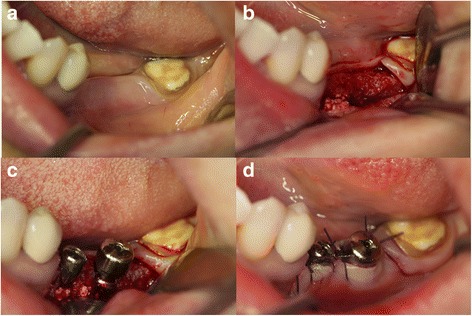
Intraoral photographs of the bone graft site at the time of implant placement (one-stage procedure). **a** Four months after the bone graft. Good gingival healing is observed. **b** Periosteal elevation to expose implant placement site. **c** Two implants (#35, 36) are inserted in a previously grafted edentulous site and healing abutments are connected. **d** Simple interrupted sutures

In 25 graft sites, a total of 39 dental implants were installed. Twenty seven implants (non-submerged 4, submerged 23) were installed immediately at the time of bone graft with the flapless technique, while 12 placements (non-submerged 2, submerged 10) were delayed in average of 5.1 months (Fig. 2). Depending on available width of the crestal bone ridge, screw-type titanium implants of 3- to 5-mm diameters with at least 8-mm length were chosen for the placement. Crest modules of the implants were placed approximately 0.5 mm below the alveolar ridge, and all implants were placed with torque between 30 and 40 Ncm. The implant-placement protocols and drilling sequence followed descriptions provided by the manufacturer’s surgical manual. The primary Implant Stability Quotient (ISQ) was measured using a resonance frequency analyzer (Osstell™, Goteborgsvagen, Sweden). After the placement, healing abutment was connected on implants inserted in a one-stage protocol, while cover screw was connected on implants inserted in a two-stage protocol. After average of 4.1 months of healing, the first impression taking and abutment connection were performed. At the time of abutment connection, secondary ISQ was measured and implant integration was clinically checked. Final prostheses were delivered in average of 2.8 months. All prosthodontics procedures were carried by a highly experienced prosthodontist, and the clinical prognosis was recorded by observation of the peri-implant tissue at each visit (Table 2).

**Table 2:** Number of implants by surgical protocols

Surgical timing	1-stage vs. 2-stage protocol	Number
Immediate (flapless) implant placement	1-stage, non-submerged	4
	2-stage, submerged	23
Delayed implant placement	1-stage, non-submerged	2
	2-stage, submerged	10
Total		39

The following success criteria for implants were used: (1) no persistent or irreversible subjective pains/complaints, (2) no recurring peri-implantitis infection or suppuration, (3) no perceptible mobility, and (4) no continuous radiolucency around the implant-to-bone contact. The implant was considered a failure if more than 1 mm of bone resorption was found after 1 year of the loading. The failed implants were still included in a survival rate if they were not physically removed. The follow-up period varied from 8 to 68 months (mean 33.7 ± 18.3 months) after the bone augmentation.

### Measurement of alveolar crest change

All patients took panoramic and standardized periapical radiographs pre- and postoperatively to determine the severity of ridge resorption. The paralleling technique was used in intraoral radiography for both linear and dimensional accuracy. One year after the ridge augmentation, the crestal bone loss around the implant was measured by taking the follow-up radiographs. Since conventional radiograms could only provide information on vertical resorption of the ridge, cone-beam computed tomography (CBCT) was additionally taken to allow three-dimensional comparison of alveolar bone width changes. Two experienced radiological technologists filmed all radiographs.

The baseline radiographs were taken before and immediately after the surgery and then compared with radiographs taken at 12 months after the graft to evaluate alveolar crest changes. Radiographs were inspected independently by a single evaluator to identify any bone loss.

Distances between implant shoulder and the first visible bone-implant contact (mm) were measured using PACS software (INFINITT PACS 3.0.9.1, Seoul, Korea). The clinician scored two marks designating where the crestal bone intersected the implant body as shown on software. Mesial and distal bone losses of the implant were measured to calculate the mean marginal bone loss. Change in crestal bone height of each implant was calculated from the differences between the initial and final measurements from standardized periapical radiographs. The magnification rate was taken into consideration to compensate proportional differences between the real implant length and the length shown on the radiographs. Each gap between threads of implant fixture was 1 mm, and this known implant dimension was used as a dimensional reference in evaluating observed alveolar bone loss in radiographs. Measurements were rounded to the nearest 0.01 mm.

## Results

A total of 39 implants were placed in 25 patients who were treated with minimal invasive ridge augmentation technique for the placement of implants during 2009 to 2014. Of the patients who had taken follow-up CBCT, analytic descriptions regarding the increase in alveolar bone volume obtained at the graft sites at the time of bone graft and implant placement are shown in Table 2; mean volume of the ridge augmentation obtained and percentage of reduction after implant loading were calculated.

**Fig. 3:**
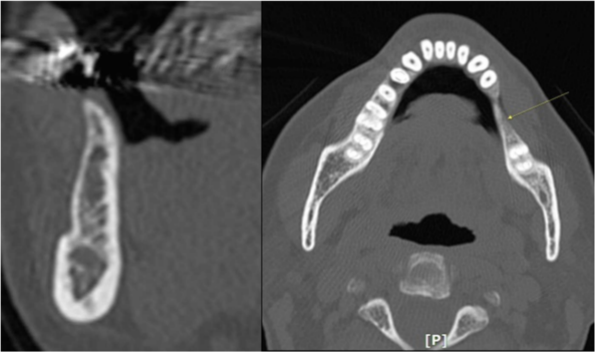
CBCT, preoperative view. The alveolar bone of #35, 36 (tooth loss state) was deficient severely. It needed bone graft to increase the width of alveolar bone where implant could be placed

**Fig. 4:**
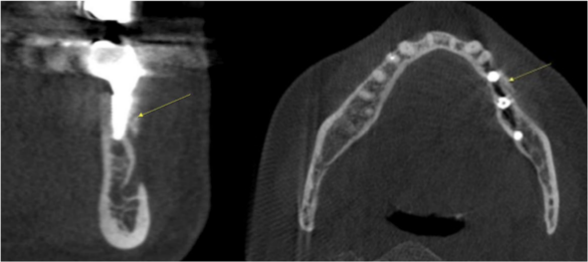
CBCT, 2 years after the restoration. The alveolar bone where bone graft using the tunneling technique is marked by a *yellow arrow*

A 58-year-old female patient took CBCT before and after bone graft, and the width of alveolar bone increased 4.32 mm added to 4.6 mm of former alveolar bone width (Fig. 3). After 4 months, the patient received one-stage implant placements (#35: 4.0D/8L, #36: 5.0D/8L) on the bone graft site. Two years after the GBR, the re-measured width of alveolar bone was 8.13 mm, and this suggested that the resorption rate of bone graft material was 18.3 % during 2 years, which was an adequate clinical prognosis (Fig. 4).

### Measurement of implant stability

Primary and secondary ISQ scores (RFA values) of 39 implants did not show statistically significant differences (Table 3). In general, secondary ISQ showed higher scores than primary ISQ in all implant sites suggesting that osseointegration was stabilized over the period of the healing process.

**Table 3:** Mean primary and secondary implant stability quotient (ISQ)

Implant sites	Number	Primary ISQ	Secondary ISQ	*P* value
Mx. anterior	28	63.7 ± 9.5	66.9 ± 14.1	NS
Mx. posterior	7	65.0 ± 12.2	69.7 ± 8.4	NS
Mn. posterior	4	74.8 ± 5.9	78.3 ± 6.7	NS
Total	39	65.1 ± 10.1	68.6 ± 12.9	NS

Statistically significant (*p* < 0.05). *P* values (Wilcoxon signed-rank test)

*NS* nonsignificant

### Clinical findings

As for postoperative complications, four graft sites showed infection symptoms, like swelling and tenderness during the treatment. The other one had buccal fenestration, and secondary bone graft was performed by the same technique. Keratinized gingiva was preserved at all sites and incision sites re-epithelialized completely within 1 month. Implants were successfully integrated except in one graft site. Out of 39 implants, 37 implants (94.9 %) were successful and 38 implants (97.4 %) survived to date according to the Albrektsson success criteria.

## Discussion

In oral and maxillofacial surgery, vertical or horizontal bone augmentation is often necessary to establish adequate bone volume especially when placing dental implants in partial or complete edentulous patients. Resorption in alveolar ridge may interfere with the insertion of dental implants.

To reconstruct adequate bone volume in alveolar bone width deficient area, three-dimensional bone overfilling is required since bone graft materials undergo gradual resorption in both width and height. Several bone grafting techniques and novel bone graft materials have been devised to accomplish this, including conventional horizontal ridge augmentation methods of using either or both particulate and block bone graft materials in guided bone regeneration (GBR). A major complication with conventional ridge augmentation technique, however, is early membrane exposure, which causes infection that result in severely compromised bone regeneration. Often occurring wound dehiscence is also a problem in sites where esthetics is of major concern. Therefore, intact primary wound coverage is mandatory for a good prognosis.

In clinical practice, implants placed in augmented ridges have a high rate of failure. Chiapasco et al. reported survival rates of implants ranged from 92 to 100 % for GBR, from 60 to 100 % for onlay bone grafts, from 91 to 97.3 % for bone splitting for ridge expansion, from 90.4 to 100 % for distraction osteogenesis, and 88.2 % for revascularized flaps [9]. In another report, the same author commented that overall survival rates of implants placed in augmented ridges range from 60 to 100 %, with a mean of 87 % [10].

According to a systemic review by Lang et al., implants placed immediately into fresh extraction sockets after at least 1 year showed a 2-year survival rate of 98.4 % (97.3–99 %), and this rate was close to 97.4 % reported in this paper [11]. Particulate and block bone grafts did not show statistically different volumetric changes after the augmentation [12].

Block and Degen had reported that particulate human mineralized bone can be successfully used to augment the thin posterior mandibular ridge through the minimal invasive horizontal ridge augmentation (MIHRA) method introduced in this report [13]. There are several advantages of the MIHRA technique compared to conventional bone augmentations. The MIHRA method is relatively less morbid and less technique-sensitive, and it does not require flap elevation or membranes. Absence of flap elevation ensures better preservation of keratinized gingiva, and adequate overfilling is also possible since primary coverage is not directly required. Moreover, minimal invasive procedures bring forth minimal implant exposure and infection that result in good mechanical stability of bone graft material.

## Conclusions

The bone graft material retained within a pouch formed by U-shaped incision and tunneling technique resulted without much complications. The sample size of this study was small, and the augmented sites differed in location and types of bone materials used. Moreover, a control group was absent for a comparison of effectiveness of MIHRA.

Within the limitations of the study, prognosis of implants placed above the alveolar bone and regenerated bone volume showed that the MIHRA is a relatively simple, safe, and effective method of reconstructing alveolar bone ridge defects in partially edentulous patients.
